# Particle Swarm Optimization and Salp Swarm Algorithm for the Segmentation of Diabetic Retinal Blood Vessel Images

**DOI:** 10.1155/2022/1936482

**Published:** 2022-08-23

**Authors:** Liwei Deng, Shanshan Liu, Xiaofei Wang, Guofu Zhao, Jiazhong Xu

**Affiliations:** ^1^Heilongjiang Provincial Key Laboratory of Complex Intelligent System and Integration, School of Automation, Harbin University of Science and Technology, Harbin 150080, China; ^2^Key Laboratory of Advanced Manufacturing and Intelligent Technology Ministry of Education, Harbin University of Science and Technology, Harbin 150080, China; ^3^Jiamusi Branch of Heilongjiang Academy of Agricultural Machinery Sciences, Jiamusi 154003, China

## Abstract

In recent years, the incidence of diabetes has been increasing year by year. Since most of the fundus lesions are located near blood vessels, the image information is complex, and the end vessels are difficult to identify. So, a new segmentation method of diabetic retinal vessel images based on particle swarm optimization and salp swarm algorithm is proposed. This paper uses a Gaussian filter to enhance the main blood vessels, and a top-bot hat transform is used to strengthen the end vessels. The preprocessing process is completed by combining and reconstructing the two images through a normalization operation. The improved particle swarm optimization and salp swarm algorithms perform multi-threshold segmentation on the preprocessed vessel images. The best fit value, Structural Similarity Index Measure, Peak Signal to Noise Rati, feature similarity index measure, sensitivity, accuracy, regional consistency, Dice coefficient, Jaccard similarity, and Shannon entropy are selected for comprehensive evaluation and analysis. The results showed that this paper's improved particle swarm-salp swarm algorithm for segmenting diabetic retinal blood vessel images is more efficient, and the threshold is better. The vascular segmentation method in this paper is applied in medical image processing, which improves the accuracy of medical image processing and reduces the computational effort.

## 1. Introduction

Diabetic retina (DR) is one of the four significant major cures for blindness worldwide. Studies have shown that the rate of advanced retinopathy blindness in China and India has been as high as 37.4% [1]. The blood vessels inside the eye are densely distributed. Therefore, eye lesions in most diabetic patients are associated with blood vessels.

The segmentation of DR vascular images is relevant to the doctors' diagnosis of the grade of retinopathy in diabetic patients and the subsequent treatment. Current fundus vascular segmentation methods can be divided into two main categories: supervised learning and unsupervised learning. Supervised learning often requires an extensive training set, where a training model is learned several times based on known images of the vascular pixel class, and the resulting model is then used to split DR vascular images of unknown vascular pixel classes. To make the segmentation of fundus vessels more transparent, many scholars have proposed many neural network-based vessel segmentation methods. Xie proposed a method for segmenting fundus vessels using deep learning to achieve automatic extraction of vessel features and improve the accuracy of the algorithm [2]. Xue et al. proposed an improved U pattern network segmentation method for DR images, which improved the unclear situation of low-contrast vascular images [3]. The above cases are all supervised learning ways and have high data requirements, while unsupervised learning split ways have lower data requirements. Unsupervised learning does not require an extensive training set, making it easier to achieve with lower hardware requirements for the operating platform. Unsupervised learning focuses on segmenting images according to the distribution pattern of vascular image pixels. Li et al. proposed method is matching filtering of fundus vessels using line operators to make the vessel pixels more prominent and Fan Linlin's segmentation method is based on matching filtering to preprocess fundus vessel images and then doing erosion and expansion on the resulting images, which improves the accuracy of the algorithms segmentation to a certain extent [4, 5]. Fan proposed method of improving the accuracy of fundus vascular image segmentation using a combination of minimum wave transform and Gaussian filtering [6]. Along with the development of artificial intelligence, image processing technology has become a popular research direction. Rajinikanth et al.proposed the FCI evaluation procedure using the Spider Monkey Optimization Algorithm (SMOA) [7].

Traditional threshold segmentation methods are unsupervised learning and are mainly based on the OTSU algorithm, the maximum variance between classes criterion for splitting images. The single-threshold OTSU segmentation method is easy to implement when dealing with images with simple information. However, due to the dense distribution of blood vessels in the fundus and the redundant data, the single-threshold segmentation method does not meet the project needs, so the single-threshold OTSU segmentation method needs to be extended to a multi-threshold field. When segmenting DR vascular images based on multi-threshold, OTSU is computationally complex and not easy to implement clinically. To address this problem, many scholars have proposed using population optimization algorithms to solve multi-threshold problems. Nasiri proposed a method for multi-threshold segmentation of fundus vascular images using the Whale Optimization Algorithm (WOA), making multi-threshold segmentation of fundus vascular images easier to implement in clinical practice [8]. Arnay uses Ant Colony Optimization (ACO) to segment fundus images with multi-threshold, and ACO's unique pheromone update method improves the segmentation accuracy of fundus vascular images [9]. Alrawi and Karajeh use Genetic Algorithm (GA) in the evolutionary algorithm to segment the matched filtered fundus image of DR after preserving more details of blood vessels [10]. Rajinikanth et al. proposed a machine-learning system (MLS) to detect the AMD using the fundus-retinal-images [11]. The unique optimization approach of the population optimization algorithm makes it more suitable for solving complex problems such as multi-threshold segmentation of DR fundus vascular images.

The main focus of this paper is to preprocess DR fundus vascular images using Gaussian matched filtering (GMF) and top-bot hat transforms, respectively, and then combine the two resulting images. Particle swarm optimization (PSO) is used to generate the parameters c_2_ of the multi-salp swarm algorithm (MSSA) based on the discrete criterion to obtain the improved particle-swarm-multi-salp swarm algorithm (PMSSA). Finally, the multi-threshold segmentation of the pretreated DR fundus vascular image after the improved PMSSA is used. The improved PMSSA segmentation of DR fundus vascular images has higher accuracy, sensitivity, and specificity. The algorithm has better convergence performance and is less likely to fall into a local optimum. To evaluate the performance of the improved PMSSA multi-threshold segmentation of DR fundus vascular images more comprehensively, a comprehensive analysis of PMSSA and same category population optimization algorithms, WOA, Social Spider Optimization (SSO), PSO, and MSSA in terms of appropriate optimal stress values Structural Similarity Index Measure (SSIM), Feature Similarity Index Measure (FSIM), Peak Signal to Noise Ratio (PSNR), Dice coefficient(Dice), Jaccard similarity coefficient (Jaccard), sensitivity, accuracy, Shannon entropy, and regional consistency was done. The results show that the DR fundus vascular image segmentation method in this paper is more accurate and retains more vascular details. The PMSSA has better iterative performance and higher stability.

The contributions of the present work are summarized as follows:

1. In this paper, we propose a segmentation method of diabetic retinal vessel images based on particle swarm optimization and salp swarm algorithm.
2. Enhanced end-vessel based on matched filtering with top-bot hat transform.
3. Multi-threshold segmentation of preprocessed DR fundus vascular images using the improved PMSSA, comprehensive analysis of the performance of the algorithm.

The main purpose of this paper is to segment the DR fundus vascular images more efficiently and accurately, retain more vascular details, and facilitate physicians' doctors to diagnose the level and diagnosis of retinopathy in diabetic patients.

## 2. Related Work

The fundus is rich in vascular tissue, and most DR fundus lesions occur near the vascular tissue, so the extraction of retinal vessels is very important in the process of DR fundus diagnosis. Recent work on retinal vascular extraction is mostly based on color fundus imaging, and RGB color fundus imaging is the only noninvasive, direct, and nondestructive way to visualize vascular tissue. Due to individual differences and different imaging environments, the differences in brightness and color of RGB images make it difficult to segment the end blood vessels in RGB images, and a variety of blood vessel segmentation methods have been developed to solve this difficulty.

In the Retinopathy Online Challenge database, Hatanaka et al. proposed a method with high generalization capability based on three microaneurysm detectors for automated microaneurysm detection [12]. Wen et al. proposed the use of principal component analysis and machine learning methods to detect microaneurysms and improve the performance of detection [13]. Dai et al. proposed an automatic detection method for MAs in color fundus images based on gradient vector analysis and class imbalance classification, which improved the sensitivity of detection [14]. Kadry et al. proposed a Computer-Aided-Procedure (CAP) to extract the blood vessel [15].

In summary, the segmentation of retinal vessel images has achieved certain results, but there is still room to improve the segmentation accuracy of retinal vessel images. Therefore, a vessel extraction method based on the improved particle swarm optimization and salp swarm algorithm is proposed in this paper to improve the accuracy of DR vessel segmentation.

## 3. Materials and Methods

### 3.1. Image Preprocessing

**Figure 1:**
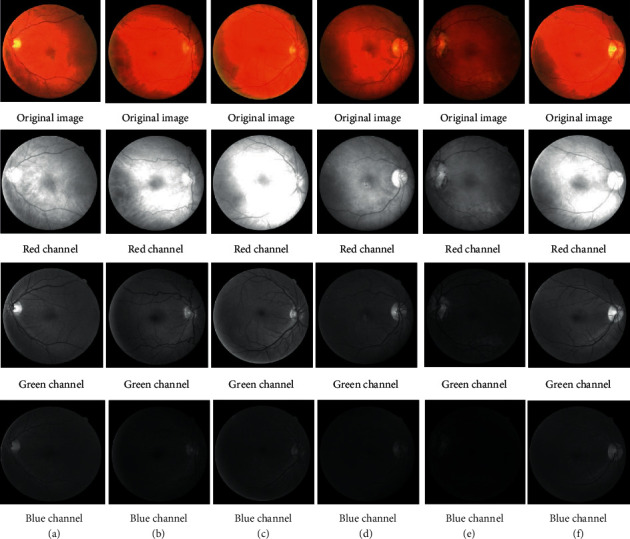
Extraction of RGB channels from color fundus image.

**Figure 2:**
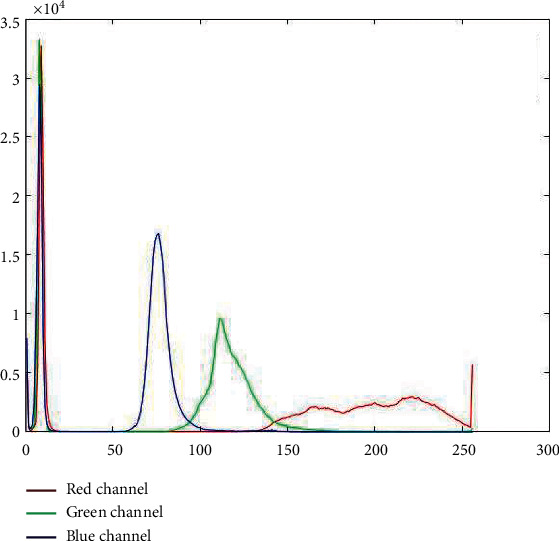
Histogram values of the RGB channels in the color original image (a).

The RGB channel extraction operation was done on the retinal images in the given DRIVE dataset to obtain the red channel, green channel, and blue channel, respectively, and the results are shown in Figure 1. For the color original image (a), the *R*, *G*, and *B* channel histogram values are shown in Figure 2. Studies have shown that the vascular information is most apparent in the green channel of DR fundus vascular images, so all studies in this paper were carried out on green channel images.

#### 3.1.1. Gaussian Match Filter

**Figure 3:**
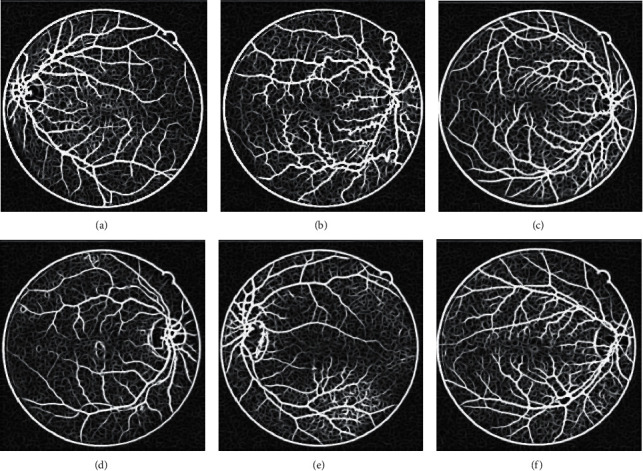
Matches the filtered green channel of DR image.

The experiments in this paper are based on GMF enhancement of the vascular portion of the green channel images [16]:

(1) $$\begin{array}{c} K_{\theta }\left(x,y\right)=-\exp\left(\frac{-x^{2}}{2\sigma^{2}}\right)y\leq \frac{\mathrm{Length}}{2}0\leq \theta \leq \pi , \end{array}$$

where *σ* is the retinal vessel scale, Length is the vessel segment length, and *θ* is the direction of filter growth in the vessel. The matched filtering results are shown in Figure 3.

#### 3.1.2. Top-Bot Hat Transformation

Top-bot hat operations do open and close operations on DR images, respectively. The top-hat process amplifies and preserves the high-frequency part of the image, while the bot-hat process preserves the low-frequency portion of the image. The closed process involves expansion followed by erosion and the open process involves erosion followed by expansion, neither of which changes the image size [17]. The specific definitions are as follows:

(2) Imgtop=Img−Imgob,Imgbot=Img−Img•b.

**Figure 4:**
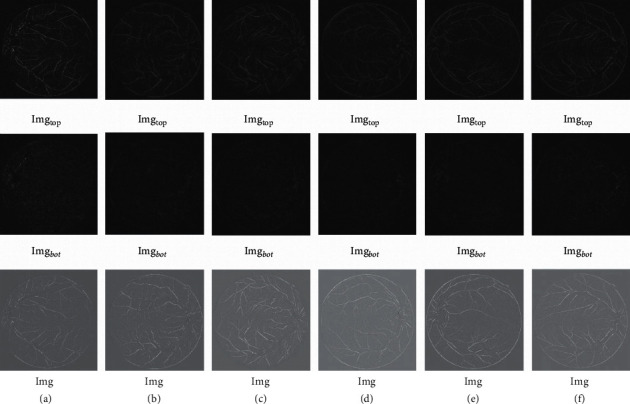
Top-bot hat transformation of the DR image.

The difference operations between the top-bot hat of the DR image make the pixels belonging to the ends of the tiny vessels in the DR vessel image more prominent, defined as follows:

(3) Img=Imgtop−Imgbot,

where Img∘*b* is the open process on the image, Img•*b* is the closed process on the image, and b is the structural element of the open and closed processes.  Figure 4 shows the results of a top-bot hat transformation performed on the DR vessel images.

## 4. Segmentation of Retinal Vessels Based on PMSSA Multi-Threshold Algorithm

Salp is an almost transparent marine invertebrate. In 2017, Mirjalili proposed a population optimization algorithm, MSSA, based on the foraging behavior of salp, which has features such as fast convergence and adjustable exploration and exploitation ratios [18].

In the mathematical model of the algorithm, the chain of salps is mainly divided into leaders and followers. Leaders update their position in a certain number of steps based on the current position of the food, and followers' positions are updated only about their predecessor's salp. In practice, the salp algorithm is used to explore *n-*dimensional space (*n* is the number of solutions) to store the positions of the salp group in a two-dimensional matrix *X*, the salp with the best fitness function is selected as food. The formula for updating the position of the salp is as follows.

(4) $$\begin{array}{c} \left\{\begin{array}{cc} x_{n}^{i}=F_{n}^{i}+c_{1}\times \left(\left(ub_{n}-lb_{n}\right)\times c_{2}+lb_{n}\right), & c_{3}\geq 0,\\ x_{n}^{i}=F_{n}^{i}-c_{1}\times \left(\left(ub_{n}-lb_{n}\right)\times c_{2}+lb_{n}\right), & c_{3}\leq 0, \end{array}\right. \end{array}$$

(5) $$\begin{array}{c} x_{n}^{i}=\frac{1}{2}x_{n}^{i}-x_{n}^{i-1}. \end{array}$$

Leaders update their positions according to formula (4) and followers update their positions according to formula (5). [ub_*n*_, lb_*n*_] is the bandwidth of the salp exploration space. *c*_1_， *c*_2_， and *c*_3_ are random numbers uniformly distributed between [0, 1]. In practice, *c*_3_ determines the direction of advancement of the salp; *c*_2_ determines the length of the advance of the salp; and *c*_1_ determines the weight of the salp influenced by food *F*. This means that the exploration and development ratio of the salp algorithm is adjustable, and to a certain extent it can avoid falling into a local optimum solution:

(6) $$\begin{array}{c} c_{1}=2e^{-{\left(4L/l\right)}^{2}}, \end{array}$$

where *l* is the number of current iterations and *L* is the number of total iterations.

### 4.1. OTSU Algorithm

The OTSU algorithm finds the optimal threshold in an image based on the maximum interclass variance. The image is partitioned into different pixel classes according to a particular rule, where the variance between individual pixel classes is the greatest [19]. The formula is as follows:

(7) $$\begin{array}{c} \left\{\begin{array}{c} \varpi_{0}=\sum_{m=0}^{t}g\left(m\right),\\ \varpi_{1}=\sum_{m=t+1}^{t}m\times g\left(m\right), \end{array}\right.\\ \left\{\begin{array}{c} \mu_{0}=\frac{\sum_{m=0}^{t}g\left(m\right)}{\varpi_{0}},\\ \mu_{1}=\frac{\sum_{m=t+1}^{t}m\times g\left(m\right)}{\varpi_{1}}. \end{array}\right. \end{array}$$

The maximum variance between classes is as follows:

(8) $$\begin{array}{c} \delta_{B}^{2}\left(t\right)=\varpi_{0}{\left(\mu_{0}-\mu_{1}\right)}^{2}+\varpi_{1}{\left(\mu_{1}-\mu_{1}\right)}^{2}, \end{array}$$

where *g*(*m*) is the percentage of greyscale value *m*; *ϖ*_0_ represents the foreground of the image; *ϖ*_1_ represents the background of the image.

The maximum variance between classes for multiple thresholds (*K* categories) is as follows:

(9) $$\begin{array}{c} \sigma_{B}^{2}=\sum_{j=0}^{K-1}\varpi_{j}\times {\left(\mu_{j}-\mu_{T}\right)}^{2}, \end{array}$$

where *μ*_*T*_ is the average grey level of the image.

### 4.2. Particle Swarm Algorithm

The PSO algorithm is a classical population optimization algorithm proposed in 1995, which is fast in iterations and has better global than local optimization seeking ability [20]. The particle swarm position update equation is as follows:

(10) $$\begin{array}{c} \left\{\begin{array}{c} V_{i\;d}^{k+1}=w\times V_{i\;d}^{k}+c_{1p}\times \left(P_{p}-Z_{i\;d}^{k}\right)+c_{2p}\left(P_{g}-Z_{i\;d}^{k}\right),\\ Z_{i\;d}^{k+1}=Z_{i\;d}^{k}+V_{i\;d}^{k+1}, \end{array}\right. \end{array}$$

where *w* is the weight of *V*_*id*_^*k*^, and *P*_*p*_ and *P*_*g*_ are the individual optimum and group optimum, respectively.

Since *c*_2_ in the salp algorithm is a set of random numbers located between [0, 1], which makes the algorithm less efficient, while the PSO algorithm has a fast iteration rate and the exploration ability is superior to local development ability, which means it can get the optimal solution at the early stage of solving simple problems, so the PSO algorithm is used to generate *c*_2_ of the Bottlenose Sea Sheath algorithm, thus bringing the improved PMSSA, which enhances the stability of the algorithm so that it has a higher probability of jumping out of the local optimum in the iterative process.

### 4.3. Improved PSO-Based Salp Multi-Threshold Algorithm for Segmenting Retinal Vessels

The single-salp algorithm (SSA) has fast convergence and adjustable exploration and exploitation ratios, and it is suitable for dealing with low-dimensional problems. *c*_2_ is the crucial parameter that determines the distance of the salp from the food location *F*. Traditional MSSA when dealing with multidimensional issues, where *c*_2_ is a set of random numbers in [0, 1], which makes the algorithm's performance in dealing with high dimensional issues and segmenting DR vessel images, MSSA is not superior, low exploration efficiency, and there is a possibility that the exploration of some salp individual is unnecessary. In contrast, the PSO algorithm has the characteristics of fast iteration and global search is superior to local search, so in this paper, PSO is used to generate a set of random arrays *Pc*_2_ fixed at the current number of iterations *l* and *Pc*_2_ is distributed between [0,1] according to the discrete criterion. This allows PMSSA to explore all directions uniformly around the current food *F*_*n*_^*i*^ during multi-threshold segmentation of DR fundus vascular images, making it less likely to fall into local optima where the PSO algorithm generates *Pc*_2_ with the following fitness function:

(11) $$\begin{array}{c} f_{\text{pso}}=\frac{\sum_{i=1}^{N_{\text{kaader}}}Pc_{2}^{i}-\bar{Pc_{2}}}{N_{\text{leader}}}, \end{array}$$

where *N*_leader_ is the number of leaders in the PMSSA algorithm. The location update formula for the improved PMSSA algorithm is as follows:

(12) $$\begin{array}{c} \left\{\begin{array}{cc} x_{n}^{i}=F_{n}^{i}+c_{1}\times \left(\left(ub_{n}-lb_{n}\right)\times Pc_{2}^{i}+lb_{n}\right), & c_{3}\geq 0,\\ x_{n}^{i}=F_{n}^{i}-c_{1}\times \left(\left(ub_{n}-lb_{n}\right)\times Pc_{2}^{i}+lb_{n}\right), & c_{3}\leq 0, \end{array}\right.\\ x_{n}^{i}=\frac{1}{2}x_{n}^{i}-x_{n}^{i-1}. \end{array}$$

**Figure 5:**
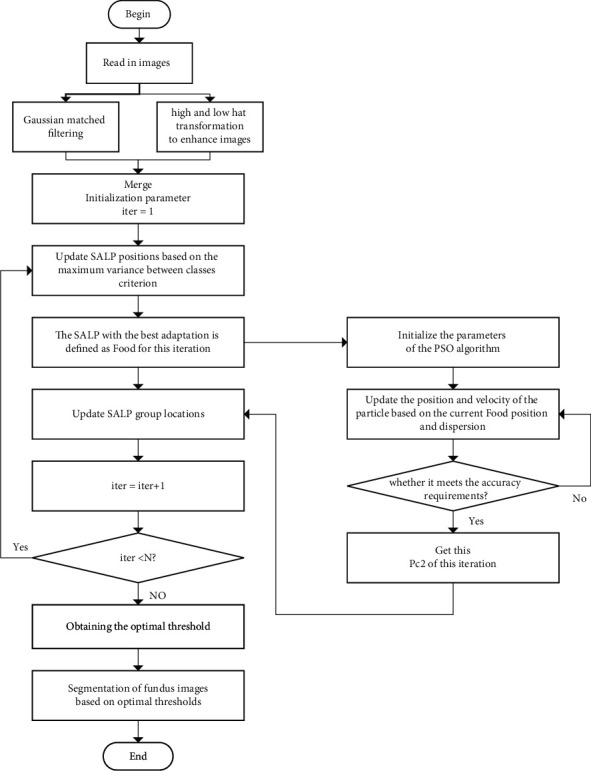
Flow chart.

**Figure 6:**
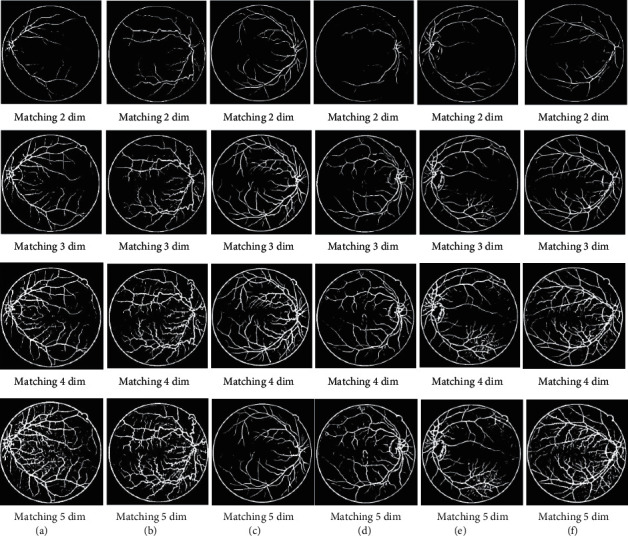
Multidimensional threshold segmentation of fundus vascular images by PMSSA algorithm.

The flow chart of the algorithm is shown in Figure 5. In this paper, the DR vessel images are processed using GMF and top-bot hat transform, respectively, and then combining the two. The results of the preprocessed DR vessel images using PMSSA multi-threshold segmentation are shown in Figure 6.

## 5. Results and Discussion

**Figure 7:**
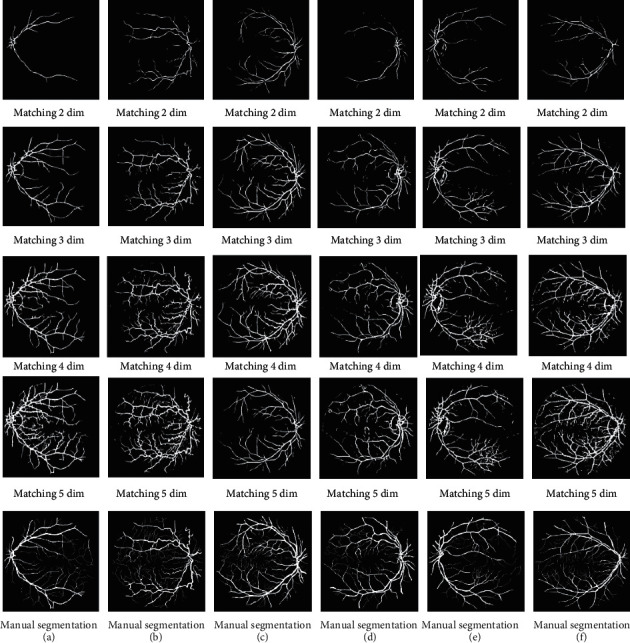
Final segmentation result.

It was observed that the 5-dimensional segmentation yielded the most abundant blood vessels. To scientifically investigate the performance of the improved PMSSA multi-threshold segmentation of DR vascular images, several classical population optimization algorithms: MSSA, WOA proposed in the literature [21], social spider optimization (SSO) proposed in the literature [22], PSO, multi-threshold segmentation of DR images in the DRIVE dataset, are selected for cross-sectional comparison experiments with the algorithms in this paper. The final segmentation result is shown in Figure 7.

### 5.1. Evaluation Indicators

In this paper, we evaluate the quality of DR vessel fundus images after algorithm segmentation based on image quality evaluation indicators: PSRN, SSIM, FSIM, Dice, and Jaccard [23–26]. The accuracy of segmentation was evaluated based on sensitivity, specificity, and accuracy [27, 28]. The bias of the algorithm segmentation was assessed based on Shannon entropy and regional consistency [29, 30].

#### 5.1.1. PSNR

PSNR is the input-to--to-output signal-to-noise ratio and is mainly used to evaluate the loss rate of an image at the signal level. When segmenting an image, a portion of the target information is eliminated while nontarget pixels are removed. Thus the evaluation indicator PSNR is introduced to evaluate the degree of distortion in the signal of the resulting image after segmentation by the algorithm in this paper, as follows:

(13) $$\begin{array}{c} PSNR\left(M,N\right)=10\log_{10}\left(\frac{MAX_{I}^{2}}{MSE\left(M,N\right)}\right),\\ MSE\left(M,N\right)=\frac{1}{HW}\sum_{i=1}^{H}\sum_{j=1}^{W}{\left(M_{ij}-N_{ij}\right)}^{2}, \end{array}$$

where *M* and *N*are the images before and after the segmentation process, respectively. The PSNR only evaluates the distortion rate of the image input and output, but not the structural change of the segmented image. To remedy this deficiency, the parameter SSIM is introduced in this paper.

#### 5.1.2. SSIM

SSIM is a metric that evaluates the variation of an image in terms of structure. In the field of unsupervised threshold segmentation, SSIM is a vital evaluation indicator that evaluates the deviation of an image in terms of brightness, structure, and contrast in terms of structure, which is specifically defined as follows:

(14) $$\begin{array}{c} l\left(m,n\right)=\frac{2\mu_{m}\mu_{n}+b_{1}}{\mu_{m}^{2}+\mu_{n}^{2}+b_{1}},\\ c\left(m,n\right)=\frac{2\delta_{m}\delta_{n}+b_{2}}{\delta_{m}^{2}+\delta_{n}^{2}+b_{2}},\\ s\left(m,n\right)=\frac{\delta_{mn}+b_{3}}{\delta_{m}\delta_{n}+b_{3}}, \end{array}$$

where *b*_1_=(*K*_1_*S*)^2^*b*_2_=(*K*_2_*S*)^2^*b*_3_=*b*_2_/2*K*_1_=0.01*K*_2_=0.03*μ*_*m*_, *μ*_*n*_ and *δ*_*m*_ are defined as follows:

(15) $$\begin{array}{c} \mu_{m}=\frac{1}{H\times W}\overset{H}{\underset{i=1}{\sum }}\overset{W}{\underset{j=1}{\sum }}M\left(i,j\right),\\ \delta_{m}=\frac{1}{H\times W-1}\overset{H}{\underset{i=1}{\sum }}\overset{W}{\underset{j=1}{\sum }}{\left(M\left(i,j\right)-\mu_{m}\right)}^{2},\\ \delta_{mn}=\frac{1}{H\times W-1}\overset{H}{\underset{i=1}{\sum }}\overset{W}{\underset{j=1}{\sum }}\left(\left(M\left(i,j\right)-\mu_{m}\right)\left(N\left(i,j\right)-\mu_{n}\right)\right). \end{array}$$

The formula for SSIM is as follows:

(16) $$\begin{array}{c} SSIM\left(M,N\right)=1\left(M,N\right)c\left(M,N\right)s\left(M,N\right). \end{array}$$

Analysis of the data in Tables 1 and 2shows that the improved PMSSA is less likely to fall into a local optimum solution. This advantage becomes more apparent as the dimensions increase. When dealing with images with complex information, the PMSSA algorithm has more obvious advantages.

#### 5.1.3. FSIM

SSIM is an essential parameter for evaluating the deviation of the segmented image from the structure, as image processing is targeted, pixels in different regions should have different weights assigned to them. Therefore, some scholars have proposed a structural evaluation criterion FSIM that assigns different weights to different regional pixels [25], which is specifically defined as follows:

(17) $$\begin{array}{c} FSIM=\frac{\sum_{X\in \Omega }S_{o}\left(X\right)\cdot PC_{p}\left(X\right)}{\sum_{X\in \Omega }PC_{p}\left(X\right)}, \end{array}$$

of which,

(18) $$\begin{array}{c} S_{PC}\left(X\right)=\frac{2PC_{1}\left(X\right)\cdot PC_{2}\left(X\right)+T_{1}}{PC_{1}^{2}\left(X\right)\cdot PC_{2}^{2}\left(X\right)+T_{1}},\\ S_{G}\left(X\right)\frac{2G_{1}\left(X\right)\cdot G_{2}\left(X\right)+T_{2}}{G_{1}^{2}\left(X\right)+G_{2}^{2}\left(X\right)+T_{2}}. \end{array}$$

#### 5.1.4. Dice and Jaccard

To evaluate the segmented image and ground truth in detail, this paper introduces the Jaccard index and Dice similarity.

Dice coefficient is also known as the Sorensen-Dice coefficient. Named after Thorvald Sorensen and Lee Raymond Dice, it is an ensemble similarity measure function that is usually used to calculate the similarity of two samples. Dice coefficient is pixel level, the real target (ground truth) appears in a certain area *A*, the target area of our model prediction result is *B*, then the Dice coefficient equation is as follows:

(19) $$\begin{array}{c} s=\frac{2\left|A\cap B\right|}{A+B}. \end{array}$$

Jaccard similarity coefficient (Jaccard) is used to compare similarities and differences between finite sample sets. Jaccard similarity coefficient is defined as the ratio of the size of the intersection of *C* and *D* to the size of the union of *C* and *D*, the definitions are as follows:

(20) $$\begin{array}{c} J\left(C,D\right)=\frac{\left|C\cap D\right|}{\left|C\cup D\right|}=\frac{\left|C\cap D\right|}{\left|C\right|+\left|D\right|-\left|C\cap D\right|}. \end{array}$$

Analyzing the data in Tables 3–7, it was found that the images segmented in 3 dimensions by the improved PMSSA algorithm were optimal in terms of evaluation indicators SSIM, FSIM, and PSNR. And the images segmented in 4 dimensions by the PMSSA algorithm were optimal in terms of evaluation indicators Jaccard and Dice. There is over-segmentation in both 4 and 5 dimensions, which may be because the segmented image has a larger vessel width than the expert segmentation. The vascular width of the image is larger than the expert segmentation. To verify the idea, this chapter introduces sensitivity (*S*_*e*_ ), specificity (*S*_*p*_), and accuracy (Acc) as one the evaluation indicators (see Table 8).

#### 5.1.5. Sensitivity, Specificity, and Accuracy

To evaluate the accuracy of pixel categorization in segmented images, this paper introduces evaluation indicators: sensitivity (*S*_*e*_), specificity (*S*_*p*_), and accuracy (Acc).

Sensitivity *S*_*e*_, specificity *S*_*p*_, and accuracy *Acc* are defined as follows:

(21) $$\begin{array}{c} S_{e}=\frac{T_{p}}{\left(T_{p}+F_{N}\right)},\\ S_{p}=\frac{T_{N}}{\left(T_{N}+F_{p}\right)},\\ Acc=\frac{\left(T_{p}+T_{N}\right)}{\left(T_{p}+T_{N}+F_{p}+F_{N}\right)}, \end{array}$$

where *T*_*p*_, *T*_*N*_, *F*_*p*_, and *F*_*N*_ represent the true example, the true counter-example in the predetermined vasculature, and the false positive, false counter-examples in the predetermined background, respectively.

Combining Tables 9–11 reveals that: compared to the other population optimization algorithms (mentioned above), the sensitivity, accuracy, and specificity of the improved PMSSA algorithm are improved, and the specificity and accuracy are significantly better. The PMMSA algorithm still has an inverse trend in 5-dimensional segmentation, but the PMSSA algorithm has the most minor inverse trend, PMSSA is more stable, and the DR vessel images' quality is better. The algorithm avoids the local solutions from getting by the other population algorithms, and the algorithm's efficiency is relatively good.

#### 5.1.6. Shannon Entropy

Shannon's entropy is used to evaluate the richness of the information contained in the segmented image, so the higher the entropy value, the richer the information contained in the image, which is specifically defined as follows:

(22) $$\begin{array}{c} H=-\sum P_{i}\cdot \log_{2}\left(P_{i}\right). \end{array}$$

#### 5.1.7. Regional Consistency

Multi-threshold segmentation of fundus vascular images is fundamentally based on particular rules for clustering pixels in fundus vascular images, which means that there is a certain similarity within the segmented classes and a certain difference among the classes. To evaluate the algorithm of this paper more comprehensively, this paper introduces regional consistency to evaluate the performance of PMSSA multi-threshold segmentation of fundus vascular images, which is specifically defined as follows:

(23) $$\begin{array}{c} UR=1-\frac{1}{\left|I\right|}\sum_{k=1}^{N}\frac{\sum_{s\in R_{k}}{\left(gray\left(s\right)-1/\left|R_{k}\right|\sum_{t\in R_{k}}gray\left(t\right)\right)}^{2}}{{\left(\max_{s\in R_{k}}\left(gray\left(s\right)\right)-\min_{s\in R_{k}}\left(gray\left(s\right)\right)\right)}^{2}}. \end{array}$$

Analysis of the data in Tables 12 and 13 revealed that the Shannon entropy value and regional consistency value of the PMSSA algorithm for multi-threshold segmentation of fundus vascular images soar in superiority with increasing dimensions. The higher the dimension, the more pixels are judged to be blood vessels in the DR image after segmentation. Tables 12 and 13 show that each algorithm has the highest Shannon entropy value in 4 and 5 dimensions. Therefore, the inverse growth of each algorithm in 4–5 dimensions can be judged as an over-segmentation phenomenon. In summary, the 3-dimensional segmentation is the closest to the expert manual segmentation. The indicators of the final result are shown in Table 14.

Combining the above evaluation indicators, it can be found that the specificity and accuracy of this paper for extracting diabetic RGB vascular images are particularly outstanding, which can reach 98.58% and 95.53%, respectively. After GMF and top-bot hat transformation, the improved PMSSA has higher segmentation accuracy in multi-threshold fundus vascular images. The algorithm has better stability in dimensions 2–5 with a more excellent and uniform probability of jumping out of the local optimal solution interval obtained by other algorithms. It also can be seen that the overall performance of DR vessel images segmented by PMSSA is the best, and the advantage becomes more evident as the dimension increases. Thus, PMSSA is more advantageous in processing images with complex vascular distributions like the images (*e*) and (*f*) in Figure 1.

### 5.2. Experimental Discussion

DR lesions are one of the common complications in diabetic patients in the late stage. In the early stage of DR lesions, timely diagnosis and treatment can effectively stop the deterioration of the disease. Fundus lesions are mostly found near blood vessels, with complex image information and difficult end-vessel identification. To address the problem of incomplete end vessel segmentation in diabetic RGB vascular image segmentation, we propose a PMSSA algorithm with significantly improved specificity and accuracy by using the particle swarm optimization algorithm to improve the parameters of the salp swarm algorithm.

This paper uses this algorithm to segment the RGB images after matched filtering and top-bot hat transformation and extracts the blood vessel features; the effectiveness of this paper's algorithm is verified by doing a comprehensive comparison analysis with other algorithms (MSSA, WOA, SSO, and PSO) on ten evaluation indicators: SSIM, PSNR, FSIM, Jaccard, Dice, Shannon entropy, regional consistency, accuracy, sensitivity, and specificity. GMF enhances the contrast between the vessel pixels and the background pixels in the DR vessel image. The top-bot hat transformation preserves the backbone information, and some of the details of the vessel end in the DR vessel images. The experimental results show that the method extracts complete vascular information with an average accuracy of 95.53% and a specificity of 98.58%. Compared with other algorithms, the accuracy and specificity are significantly improved. In dimensions 2–5, the algorithm is more stable, with a greater and more uniform likelihood of jumping out of the local optimal solution interval established by previous algorithms. Using the performance indicators listed above, it is clear that the overall performance of DR vessel images segmented by PMSSA is the best, and the advantage becomes more apparent as the dimension rises. When dealing with images with complicated vascular distributions, PMSSA is more advantageous.

Therefore, the PMSSA algorithm designed in this paper has excellent prospects in diagnosing and treating retinal disease images, which will help ophthalmologists diagnose retinal disease more effectively and significantly reduce the burden on doctors.

## 6. Conclusions

DR vascular images have a dense distribution of blood vessels and unclear end information about the end of the blood vessel. Therefore, this paper used GMF and top-bot hat transform to preprocess DR fundus vascular images. Combining the two resulting images enhances the main and partial terminal information of blood vessel, in preparation for PMSSA multi-threshold segmentation of DR vessel images. In this paper, an improved salp algorithm PMSSA based on the PSO algorithm is proposed, which is more efficient in the optimization process of salp individuals to avoid salp individuals from moving toward the nontarget direction in the current iteration process and improve the stability of the algorithm. The improved PMSSA multi-threshold segmentation preprocessed DR fundus vascular images were compared with MSSA, WOA, SSO, and PSO algorithms. Through comprehensive analysis, it is found that the improved PMSSA has a better iterative effect and is less likely to fall into the local optimal solution. The improved PMSSA has better overall performance when processing DR images with redundant information and no apparent obscure pixel distribution. Although the PMSSA algorithm is easier to obtain the optimal solution, the computation time of the algorithm is relatively long. Given this phenomenon, in future work, we will be optimized in terms of algorithms and the structure of the implementation of the algorithm code.

## Figures and Tables

**Table 1:** The fitness value of each algorithm (unit 10^3^).

Img	Dim	MSSA	WOA	SSO	PSO	PMSSA
(a)	2	−2.480670	−2.481083	−2.479847	−2.481224	−2.481225
	3	−2.614356	−2.614323	−2.613576	−2.614539	−2.614543
	4	−2.674589	−2.674806	−2.671684	−2.675460	−2.674912
	5	−2.702767	−2.700294	−2.698560	−2.704303	−2.704516

(b)	2	−2.394638	−2.394810	−2.394678	−2.381127	−2.394814
	3	−2.533017	−2.533559	−2.531972	−2.472683	−2.533594
	4	−2.597702	−2.597365	−2.590287	−2.595738	−2.597493
	5	−2.630164	−2.628601	−2.629274	−2.620240	−2.630615

(c)	2	−3.160819	−3.160358	−3.160341	−3.160789	−3.160820
	3	−3.312218	−3.311947	−3.309278	−3.311382	−3.312160
	4	−3.379922	−3.378839	−3.374332	−3.367413	−3.380899
	5	−3.410546	−3.433086	−3.426351	−3.397287	−3.413843

(d)	2	−1.577878	−1.577909	−1.578086	−1.578077	−1.578086
	3	−1.682871	−1.683968	−1.679325	−1.644257	−1.682938
	4	−1.732653	−1.733027	−1.732000	−1.715620	−1.733570
	5	−1.761228	−1.761072	−1.753048	−1.757668	−1.761228

(e)	2	−1.920536	−1.920536	−1.920346	−1.920500	−1.920536
	3	−2.035300	−2.035591	−2.034528	−2.034887	−2.035713
	4	−2.088062	−2.091452	−2.090908	−2.091239	−2.093922
	5	−2.119593	−2.120538	−2.121357	−2.104835	−2.124674

(f)	2	−2.378915	−2.378915	−2.377762	−2.378978	−2.378978
	3	−2.518641	−2.516851	−2.517037	−2.518437	−2.519070
	4	−2.581736	−2.581382	−2.581403	−2.568103	−2.581736
	5	−2.615915	−2.615805	−2.609539	−2.615896	−2.616446

**Table 2:** Optimal segmentation thresholds of each algorithm.

Img	Dim	MSSA	WOA	SSO	PSO	PMSSA
(a)	2	[52, 158]	[56, 164]	[57.162]	[555, 164]	[55, 164]
	3	[27, 81, 178]	[27, 86, 186]	[26.182.81]	[28, 86, 186]	[28, 85, 186]
	4	[21, 60, 125, 199]	[21, 58, 117, 204]	[22, 55, 123, 195]	[23, 125, 63, 204]	[23, 61, 125, 207]
	5	[20, 49, 93, 158, 242]	[12, 34, 75, 134, 223]	[21, 55, 85, 125, 203]	[164, 20, 93, 47, 238]	[20, 53, 106, 172, 223,]

(b)	2	[48, 151]	[49, 153]	[51, 152]	[61, 177]	[49, 151,]
	3	[29, 87, 186]	[30, 89, 183]	[30, 84, 184]	[212, 39, 144]	[28, 37, 183]
	4	[20, 54, 118, 199]	[24, 58, 112, 199]	[29, 66, 131, 217]	[106, 199, 51, 21]	[22, 62, 129, 212]
	5	[19, 46, 87, 113, 119,]	[18, 48, 104, 163, 224]	[22, 49, 86, 139, 217]	[45, 146, 245, 19, 93]	[20, 5199, 115, 228]

(c)	2	[55, 163]	[56, 160]	[55, 167]	[55, 162]	[55, 163]
	3	[30, 89, 187]	[31, 89, 187]	[31, 98, 196	[28, 185, 84]	[29, 87, 185]
	4	[22, 62, 127, 197]	[24, 72, 125, 205]	[27, 57, 120, 202]	[22, 189, 73, 117]	[23, 63, 130, 200]
	5	[17, 38, 71, 124, 200]	[1, 1, 1, 55, 163]	[1, 1, 29, 102, 219]	[75, 20, 245, 39, 158]	[21, 54, 104, 160, 502]

(d)	2	[53, 155]	[51, 152]	[53,152]	[52, 152]	[53, 152]
	3	[24, 73, 126]	[25, 78, 171]	[23,85,783]	[124, 226, 40]	[24, 74, 162]
	4	[21, 60, 126, 201]	[19, 55, 103, 183]	[23,59,114,195]	[140, 69, 25, 244]	[20, 57, 119, 202]
	5	[17, 39, 74, 126, 202]	[18, 43, 86, 141, 210]	[20,42,96,147,235]	[159, 20, 231, 48, 100]	[17, 39, 74, 126, 202]

(e)	2	[46, 139]	[46, 139]	[45, 137]	[143, 47]	[46, 139]
	3	[26, 76, 170]	[26.79, 167]	[29, 81, 171]	[178, 29, 88]	[26, 78, 166]
	4	[22, 62, 136, 225]	[20, 60, 123, 191]	[19, 55, 121, 199]	[202, 22, 50, 116]	[21, 55, 111, 189]
	5	[16, 36, 65, 119, 189]	[15, 45, 97, 171, 224]	[20, 55, 93, 148, 218]	[27, 136, 77, 178, 240]	[21, 54, 113, 178, 225]

(f)	2	[51, 155]	[51, 155]	[53, 151]	[52, 155]	[52, 155]
	3	[26, 77, 171]	[30, 78, 171]	[28, 90, 183]	[29, 85, 179]	[27.82.175]
	4	[22, 60, 123, 209]	[23, 63, 130, 209]	[25, 66, 128, 209]	[231, 124, 23, 69]	[22, 60, 123, 209]
	5	[17, 39, 77, 132, 211]	[18, 51, 93, 146, 211]	[23, 59, 94, 142, 222]	[141, 83, 19, 211, 39]	[19, 46, 87, 147, 227]

**Table 3:** PSNR of each algorithm.

Dim	MSSA	WOA	SSO	PSO	PMSSA
2	11.970	11.972	11.970	11.882	12.060
3	13.531	13.530	12.691	13.263	13.536
4	12.889	12.903	12.915	10.630	13.242
5	9.213	9.024	10.013	10.087	13.096

**Table 4:** SSIM of each algorithm.

Dim	MSSA	WOA	SSO	PSO	PMSSA
2	0.5694	0.5756	0.5758	0.5691	0.5694
3	0.6983	0.6994	0.6562	0.6851	0.7046
4	0.7073	0.6953	0.7107	0.5946	0.7049
5	0.5392	0.5469	0.5781	0.5802	0.6963

**Table 5:** FSIM of each algorithm.

Dim	MSSA	WOA	SSO	PSO	PMSSA
2	0.6636	0.6707	0.6705	0.6616	0.6641
3	0.7597	0.7585	0.7187	0.7476	0.7613
4	0.7440	0.7433	0.7421	0.6265	0.7502
5	0.5491	0.5612	0.6181	0.5933	0.7404

**Table 6:** Dice of each algorithm.

Dim	MSSA	WOA	SSO	PSO	PMSSA
2	0.4004	0.4037	0.4098	0.4002	0.4004
3	0.6062	0.6071	0.5758	0.5859	0.5997
4	0.6177	0.6089	0.6202	0.5372	0.6183
5	0.4149	0.4193	0.4375	0.4389	0.5973

**Table 7:** Jaccard of each algorithm.

Dim	MSSA	WOA	SSO	PSO	PMSSA
2	0.2503	0.2529	0.2577	0.2502	0.2503
3	0.4349	0.4359	0.4043	0.4143	0.4283
4	0.4469	0.4377	0.4495	0.3672	0.4475
5	0.2618	0.2653	0.2800	0.2811	0.4258

**Table 8:** Results of vascular segmentation.

Category		Correct division	Wrong division
Predictive category	Vascular	*T*_*P*_	*T*_*N*_
	Background	*F*_*N*_	*F*_*P*_

**Table 9:** Sensitivity value of each algorithm.

Dim	MSSA	WOA	SSO	PSO	PMSSA
2	0.6636	0.6707	0.6705	0.6616	0.6641
3	0.7597	0.7585	0.7187	0.7476	0.7613
4	0.7440	0.7433	0.7421	0.6265	0.7502
5	0.5491	0.5612	0.6181	0.5933	0.7404

**Table 10:** Specific value of each algorithm.

Dim	MSSA	WOA	SSO	PSO	PMSSA
2	0.9347	0.9358	0.9344	0.9346	0.9361
3	0.9638	0.9632	0.9614	0.9634	0.9701
4	0.9746	0.9700	0.9762	0.9683	0.9819
5	0.9835	0.9832	0.9715	0.9827	0.9858

**Table 11:** Accuracy value of each algorithm.

Dim	MSSA	WOA	SSO	PSO	PMSSA
2	0.9358	0.9369	0.9367	0.9358	0.9359
3	0.9553	0.9552	0.9365	0.9520	0.9553
4	0.9483	0.9485	0.9488	0.8854	0.9524
5	0.8301	0.8091	0.8500	0.8686	0.9476

**Table 12:** Shannon Entropy of each algorithm.

Dim	MSSA	WOA	SSO	PSO	PMSSA
2	0.1864	0.1926	0.1849	0.1850	0.1945
3	0.3604	0.3559	0.3446	0.3621	0.4447
4	0.4547	0.4245	0.5191	0.4092	0.6161
5	0.5495	0.6475	0.5777	0.6274	0.6449

**Table 13:** Uniformity of intra region (UR) of each algorithm.

Dim	MSSA	WOA	SSO	PSO	PMSSA
2	0.802977	0.802974	0.802973	0.802974	0.802979
3	0.802948	0.802949	0.802946	0.802947	0.802952
4	0.802943	0.802945	0.802944	0.802942	0.802945
5	0.802940	0.802940	0.802940	0.802939	0.802944

**Table 14:** Index values of the final results.

Methods	PSNR	SSIM	FSIM	Dice	Jaccard	*S*_*e*_	*S*_p_	Acc	Shannon entropy	UR
MSSA	13.531	0.6983	0.7597	0.6177	0.4469	0.7597	0.9835	0.9553	0.3604	0.802948
WOA	13.530	0.6994	0.7585	0.6089	0.4377	0.7585	0.9832	0.9552	0.3559	0.802949
SSO	12.691	0.6562	0.7187	0.6202	0.4495	0.7187	0.9715	0.9365	0.3446	0.802946
PSO	13.263	0.6851	0.7476	0.5372	0.3672	0.7476	0.9827	0.9520	0.3621	0.802947
**PMSSA**	**13.536**	**0.7046**	**0.7613**	**0.6183**	**0.4475**	**0.7613**	**0.9858**	**0.9553**	**0.4447**	**0.802952**

## Data Availability

This paper uses the DRIVE dataset from the Retinopathy Online Challenge (ROC) database. The ROC is a library of fundus images for DR screening competitions, made public by the University of Iowa in 2009. This study adhered to the tenets of the Declaration of Helsinki.
